# Acute generalized exanthematous pustulosis in a postpartum woman

**DOI:** 10.1002/ccr3.4462

**Published:** 2021-07-21

**Authors:** Hirokazu Toyoshima, Midori Mizuno, Motoaki Tanigawa, Hiroyuki Tanaka, Yuki Nakanishi, Shigetoshi Sakabe

**Affiliations:** ^1^ Department of Infectious Diseases Japanese Red Cross Ise Hospital Ise Japan; ^2^ Department of Dermatology Japanese Red Cross Ise Hospital Ise Japan; ^3^ Department of Respiratory Medicine Japanese Red Cross Ise Hospital Ise Japan

**Keywords:** acute generalized exanthematous pustulosis, differential diagnosis, discontinuation of the suspected drugs

## Abstract

The distribution of pustular erythema, characteristic clinical course, and pathological findings can help diagnose acute generalized exanthematous pustulosis. Clinical management should include discontinuation of the suspected drug, hydrocortisone administration, and careful follow‐up examination.

## Graphical abstract

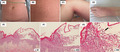

## 1 CLINICAL IMAGE

Acute generalized exanthematous pustulosis (AGEP) is a type of severe adverse drug‐related reaction. Differential diagnosis of AGEP can be challenging. A high index of suspicion is required to rapidly diagnose AGEP because of the necessity for discontinuing drugs responsible for the reaction.

**FIGURE 1:**
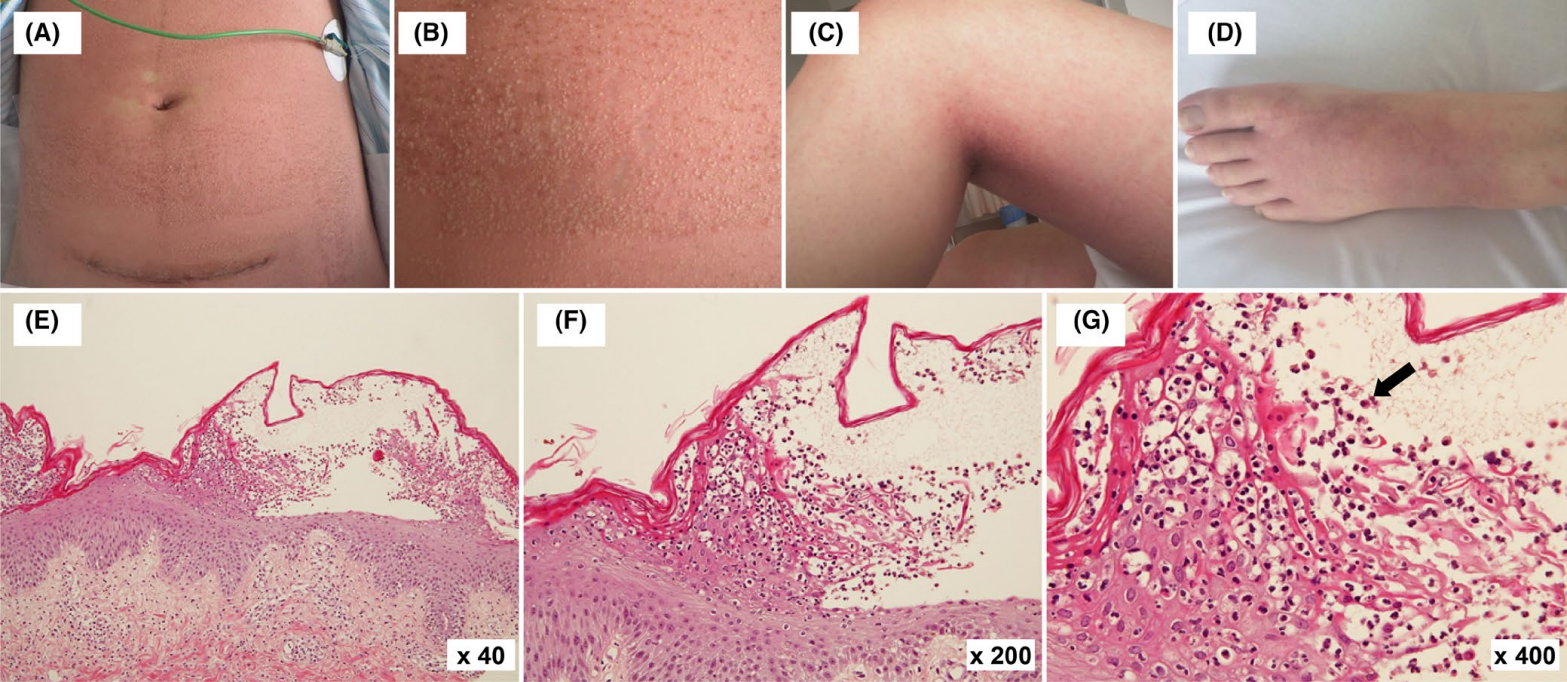
Macroscopic and histological appearance of acute generalized exanthematous pustulosis in the patient. Diffuse edematous erythema with numerous pustules on the trunk (A). Numerous tiny, non‐follicular, pustules (B) are observed when the trunk is examined closely. Erythema and several pustules are observed in the popliteal fossa (C). Mild erythema and few pustules are observed over the dorsum of the foot (D). Neutrophilic infiltration (black arrow) of the margins of the pustules is observed using hematoxylin‐eosin staining (E, ×40; F, ×200; G, ×400)

How can acute generalized exanthematous pustulosis (AGEP) be distinguished from other conditions? A 21‐year‐old healthy Japanese woman underwent a cesarean section for fetal distress. At 5 days postoperatively, she developed pyrexia and received cefdinir and acetaminophen for postpartum mastitis. At 7 days postoperatively, pyrexia persisted and she developed hypotension, tachycardia, and tachypnea, with generalized pustular erythema, particularly on the trunk (Figure 1A–D). Laboratory test results revealed the following: leukocyte count, 22,800/µl; neutrophils, 97.6%; C‐reactive protein, 22.18 mg/dl; and procalcitonin, 3.81 ng/ml. Skin biopsy was performed. She was diagnosed with AGEP based on the pustular erythema distribution and the biopsy findings (Figure 1E–G). Cefdinir and acetaminophen, the suspected causes, were discontinued, and intravenous hydrocortisone was administered. Her clinical condition gradually improved, and the pustules receded and desquamated without recurrence during the 3‐month follow‐up period.

The AGEP validation score can be used to diagnose AGEP.^1^ However, distinguishing AGEP from other conditions (Table S1) can be difficult at onset. The clinical course, distribution of the lesions, and histological findings are crucial to establish the diagnosis. Postpartum AGEP may improve with withdrawal of the causative drug alone.^2^ This case illustrates the importance of rapid diagnosis and discontinuation of the suspected drugs.

## CONFLICTS OF INTEREST

The authors declare no conflicts of interest.

## AUTHORS CONTRIBUTION

H Toyoshima contributed to the clinical management of the patient and was involved in study conception, acquisition and analysis of the data, and drafting of the manuscript. MM contributed to the clinical management of the patient and was involved in the supervision of the drafting and critical revision of the manuscript. H Tanaka and YN were involved in the study conception. TM and SS were involved in the supervision of the drafting and critical revision of the manuscript. All authors have reviewed the final draft of the manuscript and approved its submission.

## ETHICAL APPROVAL

This study was approved by the Institutional Review Board and Ethics Committee of the Japanese Red Cross Ise Hospital (Approval number: ER2021‐8).

## CONSENT FOR PUBLICATION

Written informed consent was obtained from the patient for the publication of this report and all accompanying images.

## Supporting information

Tab S1

## Data Availability

The data that support the findings of this study are openly available in [repository name e.g “figshare”] at http://doi.org/[doi], reference number [reference number].
