# Supplementary material for: Acute generalized exanthematous pustulosis in a postpartum woman
Source: Clin Case Rep. 2021 Jul 21;9(7):e04462. doi: 10.1002/ccr3.4462 (PMC8294142; doi:10.1002/ccr3.4462)
Supplement: Supplementary file 1 — Tab S1 [file CCR3-9-e04462-s001.docx]

Supporting information

Supporting Table 1. Differential diagnosis in this case

| Condition |
| --- |
| Generalized pustular psoriasis |
| Acute generalized pustular bacterid |
| Subcorneal pustular dermatosis (Sneddon-Wilkinson disease) |
| Drug hypersensitivity syndrome |
| Toxic epidermal necrolysis |
| Impetigo |
| Immunoglobulin A pemphigus |
| Miliaria |
